# Could an evaluative conditioning intervention ameliorate paranoid beliefs? Self-reported and neurophysiological evidence from a brief intervention focused on improving self-esteem

**DOI:** 10.3389/fpsyt.2024.1472332

**Published:** 2024-10-23

**Authors:** Almudena Trucharte, Valiente Carmen, Javier Pacios, Ricardo Bruña, Regina Espinosa, Vanesa Peinado, Teodoro Pascual, Anton P. Martinez, Fernando Maestu, Richard P. Bentall

**Affiliations:** ^1^ Facultad HM de Ciencias de la Salud, Universidad Camilo José Cela, Madrid, Spain; ^2^ Instituto de Investigación Sanitaria HM Hospitales, Madrid, Spain; ^3^ Departamento de Personalidad, Evaluación y Psicología Clínica, Facultad de Psicología, Universidad Complutense de Madrid, Madrid, Spain; ^4^ Center for Cognitive and Computational Neuroscience, Complutense University of Madrid, Madrid, Spain; ^5^ Department of Experimenal Psychology, School of Physchology, Universidad Compluense de Madrid, Madrid, Spain; ^6^ Health Research Institute of the Hospital Clínico San Carlos (IdISSC), Madrid, Spain; ^7^ Department of Radiology, Rehabilitation and Physiotherapy, Faculty of Medicine, Complutense University of Madrid, Madrid, Spain; ^8^ Department of Psychology, Faculty of Science, The University of Sheffield, Sheffield, United Kingdom

**Keywords:** paranoia, implicit social cognition, evaluative conditioning, magnetoencephalography, implicit self-esteem

## Abstract

**Introduction:**

Much research on the treatment of paranoia has involved cognitive-behavioural interventions that address explicit social cognition processes. However, much of human cognition is preverbal or implicit, raising the possibility that such social judgements are implicated in paranoia. One type of implicit social cognition that has been investigated concerning paranoia is implicit self-esteem with some evidence that it may be possible to change implicit self-esteem using techniques based on conditioning theory. Therefore, the primary purpose of this research is to further evaluate the potential of this approach. At the same time, as a secondary purpose, we introduce a novel way of measuring social cognition that, we argue, has utility for investigating the psychological processes involved in paranoia.

**Method:**

We conducted two proof-of-concept studies of a novel brief intervention based on evaluative conditioning, targeting implicit cognition. The first study was conducted with a large non-clinical sample, while the second study included a small series of psychotic patients. As part of our proof-of-concept evaluation of the potential of evaluative conditioning, we attempted to probe for neurophysiological changes following the intervention using magnetoencephalography in an exploratory way in the clinical sample.

**Results:**

Our results revealed that both non-clinical and clinical participants in the experimental group showed a significant change in how they evaluated themselves in the social cognition task, which could be related to the perception of social information in a less threatening way. In addition, clinical participants in the experimental group showed changes in brain activity during the social cognition task, particularly in regions involved in emotional reactivity and mentalization processes.

**Discussion:**

Our results are encouraging, suggesting that implicit cognition is manipulable, that such manipulation affects underlying neurophysiological mechanisms, and that there may be an impact on paranoid symptoms. However, much more work is required to determine whether this approach can produce meaningful clinical change and be delivered in routine clinical settings. Finally, it is important to note that we are not claiming the clinical effectiveness of our intervention, which is in a very early stage of development. Our goal here is to demonstrate clinical possibilities that warrant further investigation

## 1 Introduction

Paranoia involves the belief that the self is under threat of imminent harm due to the intentional actions of others (1) and is characterized by personal vulnerability, mistrust, and the sense of being negatively evaluated by observers (2). When severe enough to warrant clinical attention, it takes the form of persecutory delusions, “that someone, or some organization, or some force or power, is trying to harm [the patient] in some way; to damage their reputation, to cause them bodily injury, to drive them mad or to bring about their death” (3). There is therefore considerable evidence that paranoia is distributed along a continuum of severity in the general population (2) and that this continuum encompasses delusional paranoia at the severe end of the spectrum (4). These observations have prompted research to understand the psychological mechanisms involved (5, 6) and the development of psychological interventions that are designed to alleviate the suffering of people whose paranoia has become a source of clinical concern (7).

Much of the research on the treatment of paranoia has involved cognitive-behavioural interventions that address explicit beliefs about the self and others. Here we describe two proof of concept studies of a novel and yet very brief intervention, based on evaluative conditioning, that addresses implicit cognition, showing (in our first study) that it has the potential to reduce paranoid thinking in everyday life, and (in our second study with a small series of psychotic patients), is feasible as an intervention for people suffering from delusional paranoia. At the same time, as a secondary purpose of the paper, we introduce a novel way of measuring social cognition which, we argue, has utility for investigating the psychological processes involved in paranoia and other conditions that affect the evaluation of self and others.

It is important to note that we are not claiming the clinical effectiveness of our intervention, which is in a very early stage of development. Our goal here is to demonstrate clinical possibilities that warrant further investigation.

### 1.1 Social cognition and paranoia

By definition, paranoia involves reasoning about the intentions of other people and hence psychological studies of the processes involved have focused on social cognition (8).

Childhood interpersonal adversities, particularly disruptions in early bonds that typically lead to insecure attachment styles, are a well-established risk factor for paranoia in both the general population (9, 10) and clinical samples (11; see meta-analysis by 12). According to Bowlby’s attachment theory (94), early relationships with caregivers form the foundation for future interpersonal functioning. Insecure attachment styles develop in response to inconsistent or unresponsive caregiving and are characterised by negative self and other working models (13). These styles are commonly categorised into anxious attachment, marked by a negative view of the self and heightened anxiety in relationships, and avoidant attachment, marked by social withdrawal and emotional suppression (14, 15). Hence, there is evidence that insecure attachment styles mediate the relationship between childhood adversities and paranoia (10). Consistent with previous evidence implicating negative self-esteem in paranoia (16–18), these attachment styles causally precede the development of negative schemas or beliefs about the self and others, which are the defining features of paranoid thinking (19, 20). Furthermore, recent studies that use attachment security priming have demonstrated that manipulating attachment style can directly influence changes in negative self/other beliefs in individuals with paranoia, further supporting the link between attachment styles, self-other schemas and paranoid thinking (21, 22). Interpersonal mistrust, as measured by judgments of unfamiliar faces, has also been recently highlighted as a second pathway by which insecure attachment influences paranoid beliefs (23–25).

Much of the research on social cognition in paranoia has employed measures of explicit cognition, usually in the form of questionnaires. Cognitive behavioural interventions that are designed to help patients with paranoia typically attempt to manipulate this kind of cognition, for example by asking patients to evaluate their hypotheses about interpersonal interactions (26). However much of human cognition is preverbal or implicit, raising the possibility that these kinds of social judgments are implicated in paranoia. For example, there is considerable evidence that trust judgments in response to unfamiliar faces are typically very rapid – taking a few hundred milliseconds (27) – so the abnormal responses of paranoid patients to these kinds of faces suggest a bias towards assuming untrustworthiness that is too rapid to be the consequence of deliberative thought (24, 25).

One type of implicit social cognition that has been investigated in relation to paranoia is implicit self-esteem. The results of these studies have been inconsistent, with some studies finding low implicit SE in paranoid patients (e.g., 28, 29) and some not (e.g., 30, 31). This inconsistency may be due to methodological differences, such as variations in sample sizes and heterogeneity of samples, as well as limitations of the Implicit Association Test, which, despite having good psychometric properties, has been criticised because it may not fully capture the implicit self-esteem dimensions (30, 32). However, a recent meta-analysis of 22 studies found that, overall, the evidence favoured low implicit self-esteem in patients with psychosis, although this may not be specific to those with paranoid beliefs (12). One way of investigating the role of implicit self-esteem in paranoia is to manipulate it and observe the effect on paranoid thinking.

There is evidence that it may be possible to change implicit self-esteem using techniques based on conditioning theory. Hence, Dijksterhuis (33) found that subliminal evaluative conditioning in which self-related words were paired with positive words improved implicit evaluations of the self, and Baccus et al. (34) was able to achieve a similar effect simply by the repeated pairing of self-related words with a warm, smiling face. We have recently carried out a preliminary evaluation of this technique in a study with student participants selected with high paranoia scores in which the participants were randomised to a 15-minute version of Baccus’ procedure or a control intervention, (35), finding that, in those who received the evaluative conditioning intervention, improvements in self-evaluations and paranoid beliefs, measured using experience sampling diaries, were detectable in everyday life over the following week. Hence, the primary purpose of this research is to carry out a further evaluation of the potential of this approach in a large nonclinical sample, using a more ecologically valid measure of social cognition (Study 1) and, as a proof-of-concept feasibility study, in a small clinical sample (Study 2).

### 1.2 Brain alterations and paranoid cognition

Most previous research on clinical paranoia has examined the effects of psychological interventions on social cognition in people with schizophrenia using clinical measures (e.g., 36). However, any effective intervention would likely impact the neural mechanisms underlying social cognition. At present, very little is known about whether these mechanisms are abnormal in paranoid patients. However, some researchers have attempted to investigate this possibility using functional magnetic resonance imaging (fMRI). For example, Pinkham et al. (37) reported increased amygdala activation in paranoid patients during the processing of social stimuli. Blackwood et al. (38) indicated that alterations in the left inferior frontal gyrus and left prefrontal areas may support attentional and attributional deficits associated with social cognition components in paranoia. In another study, these authors also found a hypoactivation of the rostral-ventral anterior cingulate cortex and hyperactivation of different brain areas of the posterior cingulate when patients with persecutory delusions evaluated ambiguous social stimuli which were unclear as to whether or not they were related to the self; this finding was interpreted as evidence that paranoid patients process ambiguous social information as emotionally self-relevant to a greater extent than people without persecutory delusions (39). More recently, Fuentes-Claramonte et al. (40) found reduced activation near the right temporoparietal junction, a region linked to mentalization processes, while patients with persecutory delusions were performing a virtual reality social task.

Regarding electrophysiological measures, fewer studies have been conducted on paranoid symptoms specifically, finding alterations in early components (e.g., N170 and P200) related to configurational and structural processing of facial stimuli (41, 42). Still, overall, the evidence suggests that individuals with positive symptoms exhibit impairments in social cognition, particularly in the perception of emotional faces and emotion regulation (43). Two meta-analyses indicated amplitude alterations in the N100, N170 and N250 components in patients with schizophrenia while processing emotional and neutral facial stimuli (44, 45). Other researchers have found alterations in the Late Positive Potential (LPP) in schizophrenia patients, which is known to be related to emotional regulation strategies following pleasant and unpleasant images, facial expressions, or even threatening faces (46, 47).

In recent years, there has been increasing interest in exploring the neurological impact of psychological interventions to understand the mechanisms underlying disorders better and to improve therapies. For example, Kumari et al. (48) found that paranoid patients performing a social-affective task showed a significant reduction in neural activity in threat-associated brain areas (e.g., insula and inferior frontal gyrus) after a cognitive behavioural intervention for psychosis (CBTp), compared to pre-intervention activity levels. Mason et al. (45) also found that, following a CBTp intervention, paranoid patients showed a restructuring of connections between the amygdala and both the parietal lobe and dorsolateral prefrontal cortex, suggesting greater integration between higher-order cognitive systems and those involved in threat and salience, which contributed to the reappraisal of information in the social-affective task. While these fMRI neuroimaging studies provide information on the structure and function of brain areas involved in social cognition, they fail to provide insight into the temporal dynamics of neural activity occurring, for example, before and after interventions (48). Conversely, magnetoencephalography is a neuroimaging technique considered a reliable measure for capturing momentary changes in brain activation with the best compromise between the temporal and topographical levels of analysis (49).

Thus, as part of our proof-of-concept evaluation of the potential of evaluative conditioning, we attempted to probe for neurophysiological changes following the intervention using magnetoencephalography in an exploratory way. Hence, in our second study, a small number of patients underwent MEG while their social cognition was evaluated.

### 1.3 Ecologically valid measurement of social cognition

To evaluate whether interventions such as the one considered here impact social cognitive processes associated with paranoia, it is important to employ valid measures of social cognition. Traditionally, social cognition has been measured using questionnaires, which have important limitations, requiring participants to imagine themselves in interpersonal situations and then provide indications of the expected intentions of others and their own likely reactions. A more realistic approach involves using tasks or settings in which participants have to process social information online by making judgments about the self and others (8).

Taking this approach, some researchers have shown that people with paranoia, compared to nonparanoid people, make more negative appraisals of situations and people’s intentions in particular environments (50) or social scenarios created using virtual reality (51, 52). The use of realistic scenarios may also facilitate the effectiveness of therapeutic interventions for paranoid patients. Indeed, realistic scenarios involving first and third-person perspectives sometimes presented using virtual reality, have been used to study and intervene in psychological distress and social anxiety with promising results (53, 54). Some of these interventions have aimed to modify cognitive biases (55) or social inference processes (56), or have been employed in the treatment of psychotic patients suffering from paranoid beliefs (57), with several trials currently underway (58, 59). An important practical limitation of this approach is the availability of suitable virtual reality facilities. Hence, in this study, we employ a practical alternative, in which visual images are used to create scenarios that participants then interpret by making judgments from first and third person perspectives.

### 1.4 Purpose of this study

Therefore, this study had two goals. The first was to carry out a more rigorous evaluation in nonclinical participants of the impact of our evaluative conditioning paradigm on social cognitive processes which we believe are linked to paranoia. For this purpose, we developed a new, ecologically valid way of assessing social cognition which we describe in more detail below. The second goal was to determine whether the evaluative conditioning paradigm is tolerated by patients with clinical paranoia, and feasible to use in clinical settings, and to provide a preliminary, proof of concept indication of whether the intervention affects the patients’ social cognition at behavioural and neurophysiological levels.

### 1.5 Hypotheses

Regarding our hypotheses, we anticipate that (1) Higher levels of paranoia will be associated with more negative self and other evaluations in the social inference task; (2) Insecure attachment styles (anxious and avoidant) will be associated with more self and other negative evaluations in the social inference task; (3) Higher levels of depression and anxiety will correlate with more negative self and other evaluations in the social inference task; (4) Higher levels of self-esteem will be associated with more positive evaluations in the social inference task. (5) Based on previous literature, we also expect the evaluative conditioning intervention will affect the self-concept of both non-clinical and clinical participants, and this can be manifested in the social inference task, through more positive social self-evaluations after the intervention. (6) Despite the exploratory nature of this proof-of-concept study, at the neurophysiological level, we anticipate increased activation in frontoparietal areas following the intervention in the clinical sample, indicating the reappraisal of the stimuli presented in the social inference task.

## 2 Method

### 2.1 Participants

A convenience sample of 160 Spanish university students (128 females, 32 males; age: range 18-34, mean: 21.42 ± 2.71) was recruited between November 2019 and March 2020. One participant declined to participate prior to randomisation. Based on the previous findings of Espinosa et al. (35), we expected a small to moderate effect (*f* = 0.14). According to G-Power 3.1 (60), at a pre-test alpha-level of .05, 159 participants would allow a 90% chance of capturing the interaction effect in a 3 by 2 (Group by Time) mixed-effects analysis.

The experiment was advertised as a study of social cognition performance. Inclusion criteria included: aged 18 years or older, normal, or corrected vision, and no current or past involvement with secondary care psychiatric services. Eligible participants received compensation under a university credit system and ethical approval was obtained from the Faculty Ethics Committee (ref. 2019/20-017).

### 2.2 Measures

Along with the baseline measures, the socio-demographic characteristics of the participants were obtained (age, sex, civil status, nationality, and employment).

#### 2.2.1 Baseline measures

Psychosis Attachment Measure (PAM; 61) includes 16 items assessing anxious and avoidant attachment on a 4-point Likert scale. A total score is obtained by calculating the mean for each insecure style, ranging from 0 (not at all) to 3 (very much). In our study, the internal reliability for both subscales was acceptable (α = 0.79; α = 0.76, respectively).

Depression, Anxiety and Stress Scale (DASS-21; 62) is a self-report scale designed to measure depression, anxiety, and stress on a 4-point Likert scale between 0 (Did not apply to me at all) and 3 (Applied to me very much, or most of the time). In this study, we used the total scores for depression and anxiety, which were calculated by summing the scores for each subscale, ranging from 0 to 21. For this study, the internal consistency for the anxiety subscale was acceptable (α = 0.78), and good for the depression subscale (α = 0.88).

Green Paranoid Thought Scales (GPTS; 63) is a 32-item self-report questionnaire that assesses paranoid ideas on a 5-point Likert scale ranging from 1 (not at all) to 5 (totally). There are two 16-item scales. Scale A assesses ideas of social reference, whereas scale B assesses persecutory thoughts. Scores on each scale range from 16 to 80. In our study, the internal consistency was good for both subscales (α = 0.87 and α = 0.84, respectively).

Rosenberg Self-Esteem Scale (RSES; 64) is a 10-item self-report scale designed to assess global trait self-esteem. Each item is rated on a 4-point Likert scale ranging from 1 (strongly disagree) to 4 (strongly agree). A total score is obtained by summing the ratings on each item, varying from 10 to 40. In this study, the internal reliability was excellent (α = 0.90).

#### 2.2.2 State measures

Brief state version of the Paranoia Checklist (65). The three-item version of this scale was selected in this study to assess feared harm, perpetrator intent, and negative evaluations by others. Participants have to indicate to what extent each item applies to them “at the moment” on a Likert scale ranging from 0 (nothing) to 10 (a lot). In our study, the internal consistency was good (α = 0.86).

State Adult Attachment Measure (SAAM; 66), is designed to capture fluctuations in attachment dimensions in response to situational factors. The items were rated on a 7-point Likert scale ranging from 1 (strongly disagree) to 7 (strongly agree). The SAAM showed good psychometric properties with three factors (secure, avoidance, and anxious attachment). For this study, two items with the greater loading on each attachment factor were selected. In our study, the internal consistency was good for secure attachment (α = 0.84) and questionable internal consistency for anxious and avoidant attachment (α = 0.60 and α = 0.60, respectively).

#### 2.2.3 Experimental tasks

Social Inference task: Based on Western et al. (67), an experimental task was designed to assess social inference and administered using E-Prime (version 2.0) software. A professional illustrator created pictures of sixteen everyday social scenarios (e.g., cinema, bakery, hospital, etc.) in 3D. The scenarios were designed so that a person could imagine themselves or someone else entering them. Then, two different versions of each scenario with different viewpoints (i.e., angles of vision) were generated: a) from a viewpoint at eye level (16 self-scenes), and b) from a higher viewing angle (16 other-scenes). The self- and other-scenes also differed in the presence of a character in a red T-shirt, who only appeared in the other-scenes and was called Gabriel. The same random order of scenario presentation was used for all participants.

**Figure 1:**
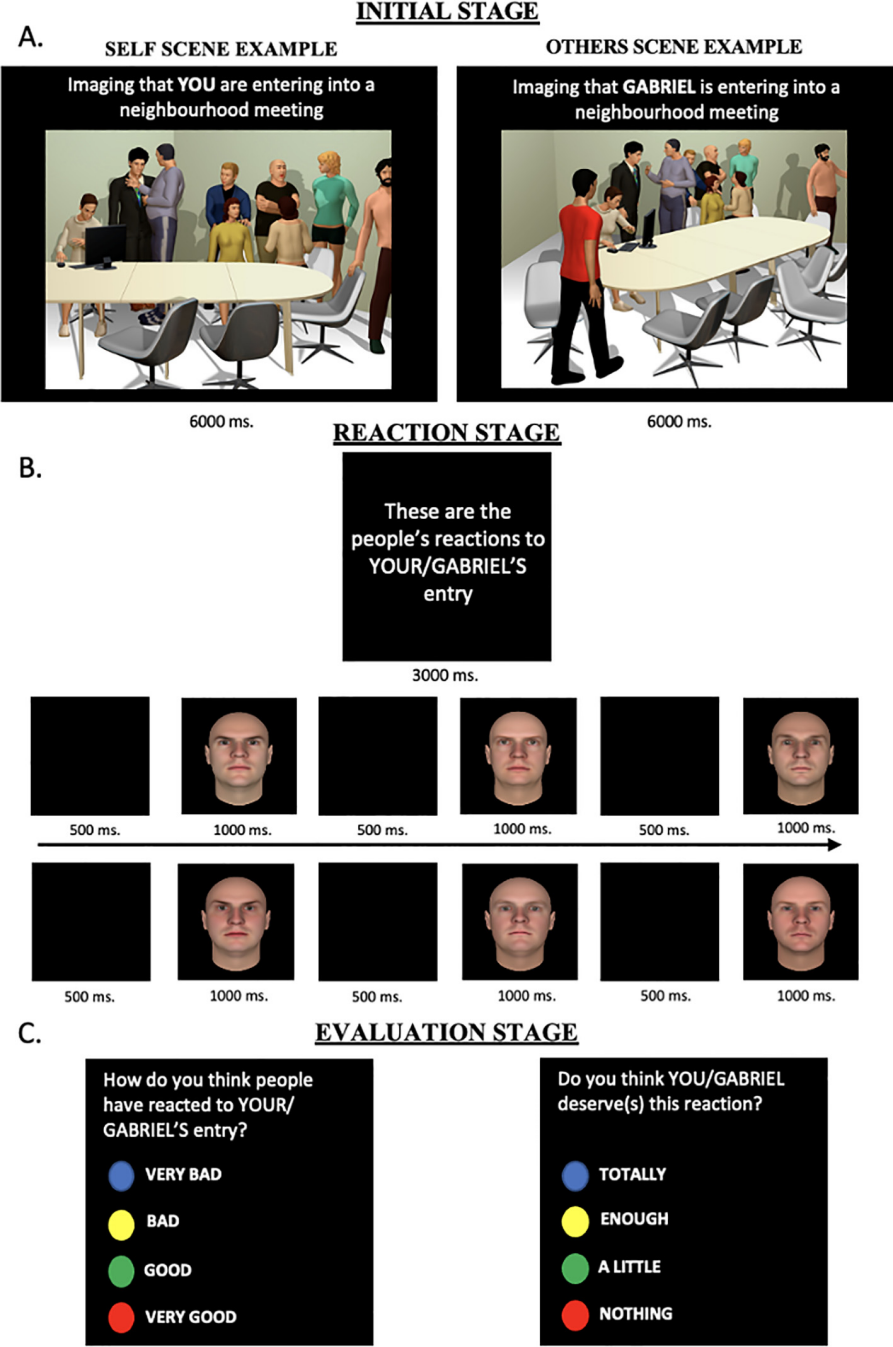
Example of social inference task for self and other scenes. The task has three consecutive stages: **(A)** Initial stage. A Self or Others scene is presented for 6000ms (12000ms for MEG experiment); **(B)** Reaction stage. Six different neutral and threatening faces were presented for 1OOOms sequentially; **(C)** Evaluation stage. Each participant rated the appraisal of the reactions and their deservedness.

As portrayed in **Figure 1**, participants were instructed to imagine either themselves or Gabriel entering each social scenario (initial stage). Next, on the reaction stage and after each scene, six computer-generated faces appeared randomly, and the participants were told these were the reactions to the entrance. The faces were selected from a pool of 50 neutral and 50 untrustworthy faces (all bald, Caucasian men) from the Princeton Social Perception Lab database (96). Once the six face trials were presented, in the evaluation stage, participants had to answer two questions: (1) Evaluation question: ‘*How do you think people have reacted to your/Gabriel’s entry?’*, ranging from 1 (very bad) to 4 (very good), and (2) Deservedness question: ‘*Do you think you/Gabriel deserve(s) this reaction?’*, ranging from 1 (totally) to 4 (not at all). The procedure was the same for all 32 social scenes and for both the pre- and post-assessment.

**Figure 2:**
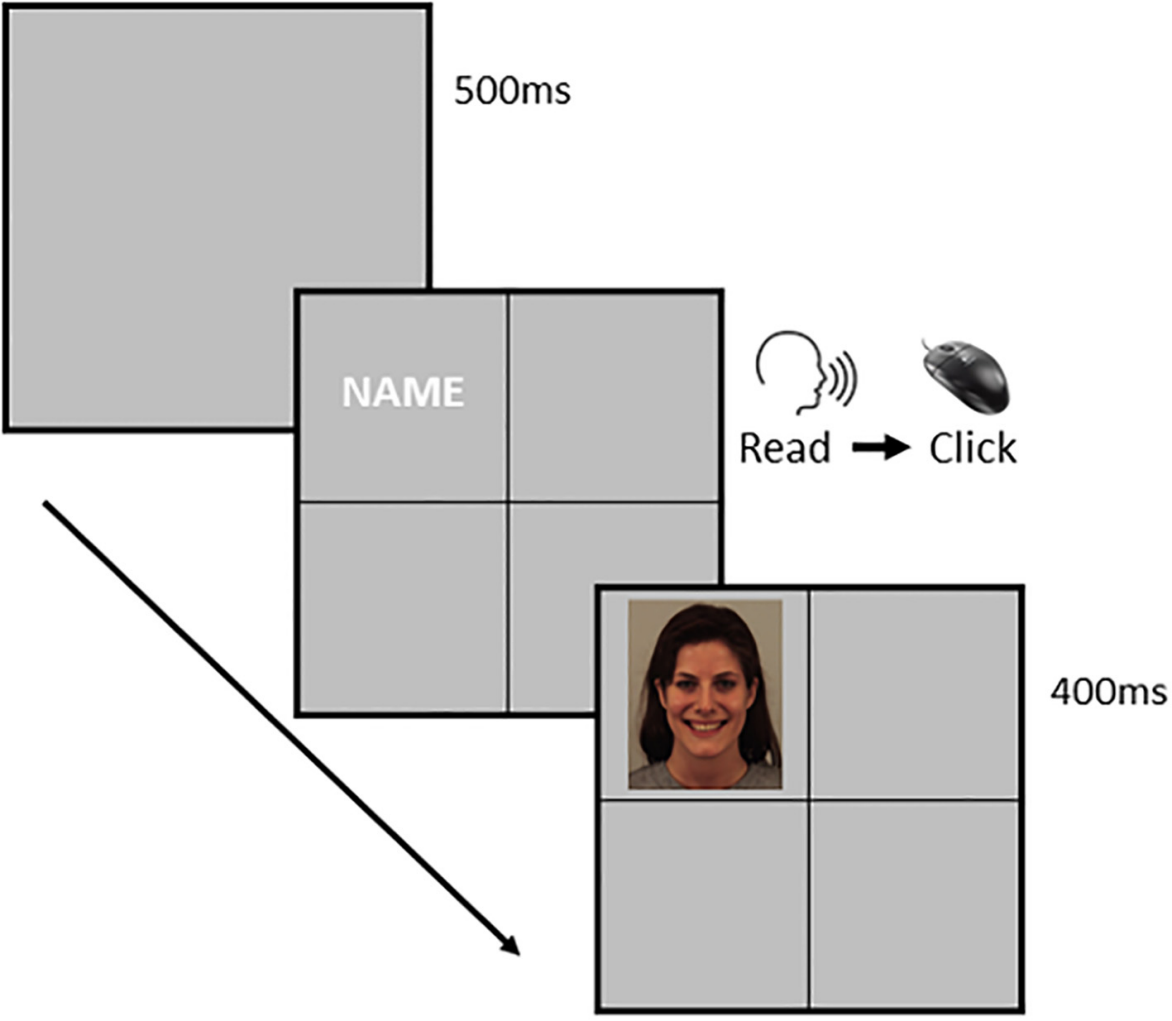
Example of classical conditioning intervention task. Example of a trial of the classical conditioning intervention. Firstly, a blank screen is presented for 500. Secondly, we present a screen divided into four quadrants. In one of the quadrants, we present words associated with the participant (first name, last name, etc.) or a control word, unrelated to the participant. They must read aloud the word and then press a button to move forward. Finally, we present a face in the same quadrant for 400with negative, neutral or positive expressions.

Classical conditioning task (CC; 34): The task was personalized for each participant with individual self-relevant information (e.g., first name, month of birth), and was presented with E-Prime software (version 2.0). The control words were personal pronouns (e.g. she/he), different first and last names and months of birth of the participants. Participants were informed that a word would appear randomly in one of the quadrants on the computer screen and instructed to read out and click on the word as quickly as possible using the mouse. In addition, they were told that doing so would cause an image to be displayed briefly (for 400 ms) in the same quadrant. This procedure was repeated for 240 trials. Self-relevant and nonself–relevant words were presented in a preprogrammed pseudorandomized order. There were three experimental conditions; 1. Positive: where self-relevant words were always paired with an image of a smiling face; 2. Negative: where they were always paired with an angry face (note. in both positive and negative conditions, the other-relevant words were paired with a random sequence of smiling, angry and neutral faces); and 3. Control: where a random selection of smiling, angry and neutral faces followed the self-relevant and non-self-relevant words (see **Figure 2**).

### 2.3 Design and procedure

**Figure 3:**
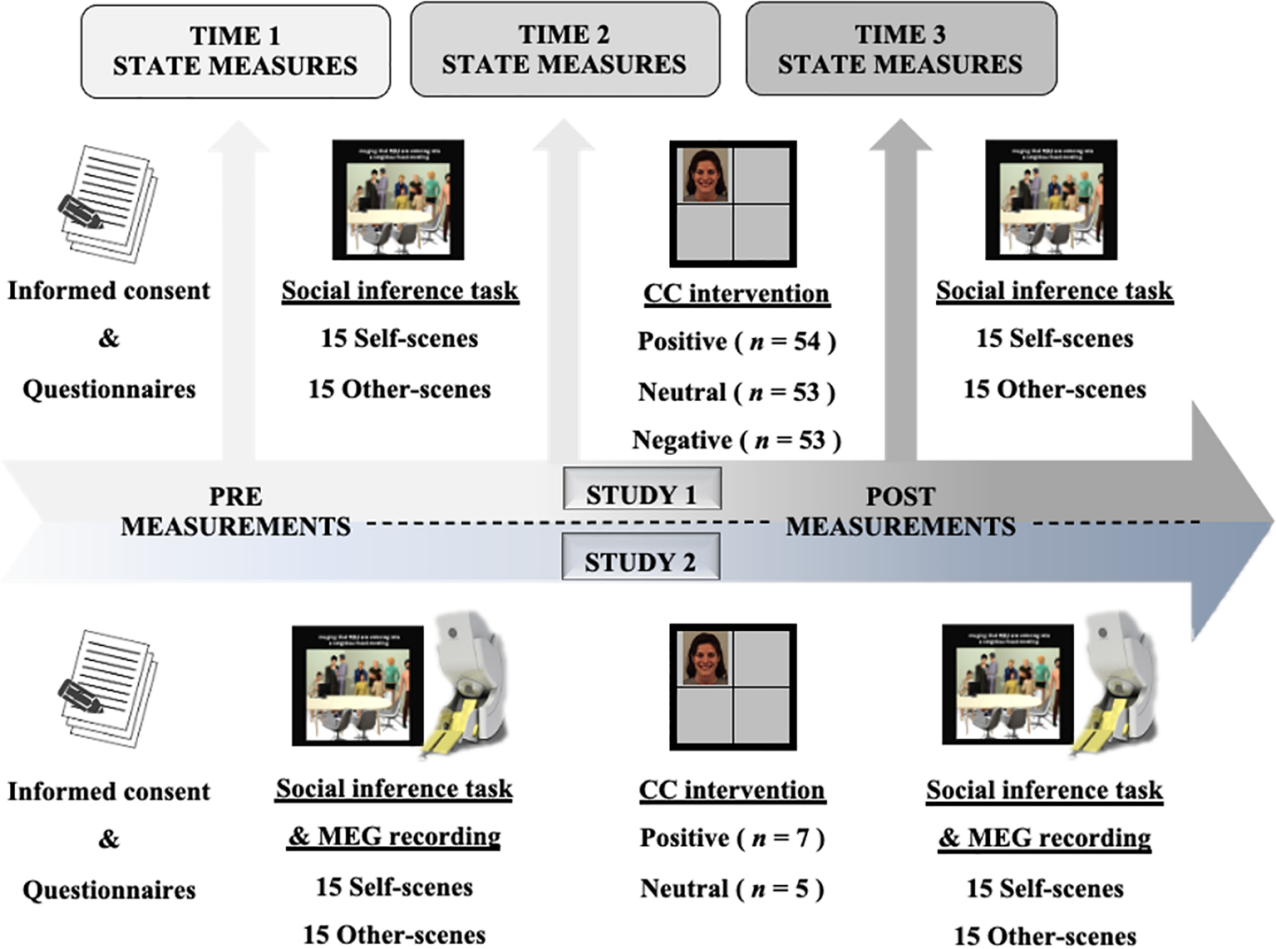
Paradigm design and procedure for both studies. The total duration of the paradigm for both studies was 70 minutes. CC, Classical Conditioning Intervention.

The study was pre-registered in OSF (https://osf.io/yfmne/?view_only=b6aef3ae82654cad9ac783e50b50fe3f). A pre-post experimental design was conducted to explore the efficacy of the CC intervention. Once participants agreed to take part in the study, they were randomly assigned— using the Markov Chain basic version software installed in Excel —to one of the three experimental conditions (i.e., positive (*n*= 54), neutral (*n*= 53), or negative (*n*= 52) CC groups) and signed the informed consent. The study was conducted in an office room with a computer desk. Firstly, participants completed the baseline measures and then performed the experimental tasks. The social inference task was performed before and after the CC intervention task. In addition, participants completed state measures of paranoia and attachment-related cognitions at three-time points: (1) before the pre-measurement of the social inference task; (2) after the pre-measurement of the social inference task and before the CC task; and (3) after the CC task and before the post-measurement of the social inference task (see **Figure 3**).

### 2.4 Data analysis

All analyses were conducted with SPSS (version 23). Firstly, the characteristics of the three classical conditioning groups were compared using the *χ^2^* test for categorical variables and the univariate ANOVA for continuous variables. Secondly, to obtain a measure of social inference, the mean scores of the evaluation and deservedness questions were calculated for self and other scenes. These scores were calculated on pre- and post-measurements for all CC groups, as well as for the total sample in the pre-stage. Thirdly, to explore the relationship of clinical measures at baseline with the social inference task, correlation analyses were conducted between these variables. Finally, to examine between-group differences related to the CC intervention, repeated measures 3x2 analyses of variance (ANCOVAs) were conducted, with a group (positive, negative, and neutral) as a between-group factor, time of assessment (pre-and post-classical conditioning task) as within-subject factor, and gender as a covariate (to compensate for possible sex effects and have better control over the random variables in the study). In addition, changes in state measures during task performance were also explored.

## 3 Results

### 3.1 Characteristics of the sample & relationship between the social inference task and baseline measures

There were no significant differences in the characteristics of the three groups at baseline (see **Table 1**). Baseline correlations showed that paranoia and anxious attachment scores were associated with less positive evaluations of both targets (self and Gabriel) in the social inference task. However, higher levels of baseline self-esteem were related to more positive self-evaluations in the social inference task. As found in previous studies, paranoid thoughts were associated with both forms of insecure attachment and low self-esteem (see **Table 2**).

**Table 1:** Demographic and clinical characteristics of group participants.

	Positive CC (*N*=54)	Neutral CC (*N*=53)	Negative CC (*N*=52)	*F/x^2^*	*p*
Demographic characteristics					
Age in years, mean (SD)	21.3 (1.63)	21.3 (2.56)	21.4 (3.28)	0.07	.92
Sex: Women, n (%)	47 (87.0)	41 (77.4)	39 (75.0)	2.70	.25
Single status, n (%)	40 (75.5)	34 (64.2)	32 (61.5)	4.38	.35
Employment, n (%)					
No employment Part-time job Full-time job	33 (62.3) 19 (35.8) 1 (1.9)	34 (64.2) 19 (35.8) 0 (0.0)	25 (48.1) 26 (50.0) 1 (1.9)	4.12	.39
Clinical characteristics					
Attachment (PAM)					
Anxious Attachment, mean (SD) Avoidant Attachment, mean (SD)	1.51 (0.59) 1.26 (0.49)	1.48 (0.49) 1.33 (0.47)	1.46 (0.66) 1.39 (0.58)	0.08 0.89	.92 .41
Symptomatology (DASS-21)					
Anxiety, mean (SD) Depression, mean (SD)	3.48 (3.57) 4.17 (4.12)	3.85 (3.79) 4.02 (3.40)	4.74 (3.92) 5.08 (5.25)	1.56 0.92	.21 .39
Paranoia (GPTS)					
Ideas of social reference, mean (SD) Persecutory thoughts, mean (SD)	25.90 (9.08) 19.67 (5.50)	24.49 (8.29) 18.92 (5.73)	25.36 (8.32) 19.60 (4.38)	0.36 0.32	.69 .72
Self-esteem (RSES), mean (SD)	30.98 (6.01)	31.47 (5.72)	31.17 (6.97)	0.08	.92

DASS-21, Depression, Anxiety and Stress Scale; GPTS, Green Paranoid Thought Scale; PAM, Psychosis Attachment Measure; RSES, Rosenberg Self-esteem Scale.

**Table 2:** Correlation values (r) between the social inference task at the pre-level and the baseline clinical measures.

Variables	(1)	(2)	(3)	(4)	(5)	(6)	(7)	(8)	(9)	(10)	(11)
(1) Self-Evaluation question	___										
(2) Self-deservedness question	-.52**	___									
(3) Other-Evaluation question	.64**	-.44**	___								
(4) Other-deservedness question	-.43**	.85**	-.63**	___							
(5) Anxious Attachment (PAM)	-.25**	.11	-.19*	.07	___						
(6) Avoidant Attachment (PAM)	-.03	.05	-.01	.04	.02	___					
(7) Anxiety (DASS-21)	-.14	-.001	.02	.02	.33**	.22**	___				
(8) Depression (DASS-21)	-.10	-.03	-.12	.02	.37**	.26**	.53**	___			
(9) Ideas of social reference (GPTS)	-.30**	.12	-.15*	.09	.49**	.17*	.43**	.41**	___		
(10) Persecutory thoughts (GPTS)	-.38**	.09	-.21**	.07	.28**	.10	.24**	.24**	.59**	___	
(11) Self-esteem (RSES)	.19*	-.02	.12	-.02	-.49**	-.19*	-.45**	-.64**	-.54**	-.29**	___

DASS-21, Depression, Anxiety and Stress Scale; GPTS, Green Paranoid Thought Scale; PAM, Psychosis Attachment Measure; RSES, Rosenberg Self-esteem Scale.

**p≤.01; *p≤.05.

### 3.2 Pre-post changes in the social inference task after the CC intervention

After controlling the effect of gender, the analysis revealed a significant *Group x Time* interaction for the Self*-*evaluation question, but not for the other*-*evaluation question (see **Table 3**). *Post-hoc* comparisons showed that participants in the positive CC group had a better appraisal of themselves after the intervention (*F* (1,53) = 8.54; *p* <.01; η²=.13). There was no effect on the deservedness question.

**Table 3:** Means, standard deviations and 3x2 repeated measures ANCOVAs statistics for study changes pre-post in the social inference task.

Social inference Task	Total (*N*=159)	Positive CC (*N*=54)	Neutral CC (*N*=53)	Negative CC (*N*=52)	ANCOVA				
	*M (SD)*	*M (SD)*	*M (SD)*	*M (SD)*	*Effect*	*F ratio*	*df*	*p*	η²
Self-evaluation question					G	0.95	2,155	.38	
Pre	2.58 (0.27)	2.56 (0.27)	2.62 (0.32)	2.57 (0.23)	T	0.10	1,155	.74	
Post	_____	2.67 (0.23)	2.61 (0.34)	2.54 (0.35)	G x T	3.32	2,155	.03*	.04
Self-deservedness question					G	0.02	2,155	.97	
Pre	2.90 (0.59)	2.87 (0.52)	2.92 (0.66)	2.91 (0.60)	T	0.01	1,155	.89	
Post	_____	2.82 (0.56)	2.92 (0.71)	2.91 (0.72)	G x T	0.33	2,155	.71	
Other-evaluation question					G	0.60	2,155	.54	
Pre	2.58 (0.31)	2.60 (0.28)	2.60 (0.36)	2.55 (0.27)	T	1.76	1,155	.18	
Post	_____	2.65 (0.28)	2.65 (0.34)	2.58 (0.33)	G x T	0.20	2,155	.81	
Other-deservedness question					G	0.14	2,155	.86	
Pre	2.88 (0.62)	2.84 (0.52)	2.85 (0.71)	2.94 (0.61)	T	0.04	1,155	.84	
Post	_____	2.78 (0.53)	2.85 (0.71)	2.90 (0.69)	G x T	0.35	2,155	.70	

There was not any significant effect of the covariate. CC, Classical Conditioning; G, Group; Time, Time; Self-Evaluation question = *‘How do you think people have reacted to your entry?’*; Self-Deservedness question = *‘Do you think you deserve this reaction?’*; Other-Evaluation question = *‘How do you think people have reacted to Gabriel’s entry?’*; Other-deservedness question = *‘Do you think Gabriel deserves this reaction?’*; M, Mean; SD, Standard Deviation; *p<.05.

### 3.3 State measures changes across the paradigm

On the paranoia state measure, there was a significant *Group x Time* interaction, after controlling the effect of gender (*p*>.1). *Post-hoc* analysis revealed that participants in the negative CC group showed a significant increase in their levels of paranoia between the first and third measures (*F* (2,49) = 3.27; *p* =.04; η²=.11).

For attachment measures, there was a marginal *Time* effect on levels of secure attachment showing that, averaged across all CC groups, on both measures there was a decrease at the second and third points of assessment (*F* (2,151) = 2.72; *p* =.06; η²=.03). In addition, there was a significant *Group x Time* effect for both anxious and avoidant attachment. *Post-hoc* analysis showed that participants in the positive CC group experienced a significant decrease in the level of anxious (*F* (2,50) = 6.61; *p* <.01; η²=.20) and avoidant attachment (*F* (2,50) = 5.42; *p* <.01; η²=.17) (see **Table 4**).

**Table 4:** Means, standard deviations and 3x3 repeated measures ANOVAs statistics for study changes in the State measures.

State measures	Positive CC (*N*=54)	Neutral CC (*N*=53)	Negative CC (*N*=52)	ANOVA				
	*M (SD)*	*M (SD)*	*M (SD)*	*Effect*	*F ratio*	*df*	*p*	η²
Paranoia								
Time 1	0.65 (1.64)	0.25 (0.83)	0.33 (0.70)	G	0.48	2,152	.62	
Time 2	0.71 (1.81)	0.50 (1.31)	0.39 (0.79)	T	1.24	2,151	.29	
Time 3	0.64 (1.69)	0.51 (1.11)	0.90 (1.71)	G x T	2.51	4,302	.04*	.03
Secure Attachment								
Time 1	5.43 (1.85)	5.44 (1.68)	5.50 (1.43)	G	0.13	2,151	.87	
Time 2	5.16 (1.81)	5.25 (1.92)	5.28 (1.53)	T	2.72	2,150	.06	.03
Time 3	5.10 (1.86)	5.10 (1.97)	5.30 (1.57)	G x T	0.79	4,300	.52	
Anxious Attachment								
Time 1	3.80 (1.68)	3.43 (1.89)	3.42 (1.82)	G	0.11	2,152	.89	
Time 2	3.42 (1.82)	3.46 (1.94)	3.60 (1.91)	T	0.32	2,151	.72	
Time 3	3.55 (1.88)	3.30 (1.88)	3.45 (1.93)	G x T	4.15	4,304	<.01**	.05
Avoidant Attachment								
Time 1	1.98 (1.50)	2.25 (1.44)	1.80 (1.72)	G	1.29	2,152	.27	
Time 2	1.71 (1.49)	2.30 (1.54)	1.91 (1.97)	T	0.31	2,151	.73	
Time 3	1.63 (1.55)	2.18 (1.49)	2.04 (1.94)	G x T	2.46	4,302	.04*	.03

There was not any significant effect of the covariate. CC, Classical Conditioning; G, Group; Time, Time; M, Mean; SD, Standard Deviation; **p<.01; *p<.05

## 4 Method

The experimental paradigm was adapted for use with a clinical population and to allow neurophysiological measurement. Given the exploratory nature of this study, we chose to use magnetoencephalography (MEG), since this technique offers the best compromise between the temporal and topographical levels of analysis (49). To our knowledge, no previous study has investigated the electrophysiological counterparts of therapeutic change induced by psychological interventions such as the one proposed here.

### 4.1 Participants

Previous studies (i.e., 35) and results from study 1 suggested that the behavioural change induced by our CC protocol would be of moderate to large size. Thus, to detect this size (*dz*= 0.6) according to G-Power 3.1 (60), at a pre-test alpha-level of.05, 20 participants would be required for an 80% chance of capturing the effect.

Although study recruitment was compromised by the SARS-Cov-2 pandemic. Fifteen men with a diagnosis of Paranoid Schizophrenia (ICD codes F20) and with a score of at least one item on the Present State Examination (PSE-10, SCAN, 19, 68) attending the psychiatric rehabilitation services of the National Health System Network were approached on the recommendation of their clinical teams. Participants were excluded if they had any condition that could affect MEG data acquisition (i.e., diagnosis of cocaine abuse disorder; habitual cannabis use; organic disorders or severe cognitive impairment; wearing a cardiac pacemaker or any metal object) and one refused to participate prior to randomization. Thus, 14 participants were randomly assigned to the positive (*n*=7) and neutral (*n*=7) CC groups. Of these, two participants in the neutral group were subsequently found to not meet the MEG inclusion criteria (drug consumption and visual impairments) and were therefore excluded from the analysis. Eligible participants received 30 € and study confidentiality was maintained throughout the procedure. Ethical approval for the study was obtained from General Hospital (ref. 16/483-E_BS) and from the institutional Review Committee of the Centre for Biomedical Technology (Technical University of Madrid).

### 4.2 Design and procedure

Participants were randomly assigned—using an automated randomisation software installed in Excel—to the experimental conditions. While the clinical assessment (baseline measures) was conducted in the rehabilitation centres, the neurophysiological measures were taken at the MEG laboratory (Centre for Biomedical Technology). Both assessments were conducted in the same week by the same member of the research team (see **Figure 3**).

### 4.3 Measures

Along with the baseline measures, the socio-demographic characteristics of the participants were obtained (age, sex, civil status, employment, nationality, and medication).

#### 4.3.1 Baseline measures

The Relationship Questionnaire (RQ; 69) is composed of four vignettes describing four attachment styles: secure, fearful, preoccupied and dismissing. Participants had to rate on a 7-point scale from 1 (not at all like me) to 7 (very much like me) how each description corresponds to their general relationship style. Following the literature (Yárnoz-Yaben & Comino, 2011), a dimensional measure of anxious and avoidant attachment was derived from scores in each of the four styles.

The Hospital Anxiety and Depression Scale (HADS; 70), is a 14-item self-report questionnaire that consists of two subscales of seven items designed to measure levels of anxiety and depression. Each item scores on a 4-point Likert scale ranging from 0 (as much as I always do) to 3 (not at all). A total score is obtained by summing the ratings on each item for anxiety and depression separately ranging from 0 to 21. In our study, the internal reliability for both subscales was acceptable (α = 0.71; α = 0.67, respectively).

Persecutory Ideation Questionnaire (PIQ; 71), is a 10-item questionnaire designed to measure persecutory ideation in clinical samples. Items are rated on a 5 Likert scale from 0 (very untrue) to 3 (very true). A total score is obtained by summing the ratings on each item, varying from 0 to 40. In this study, the internal reliability was excellent (α = 0.96).

Rosenberg Self-Esteem Scale (RSES; 64), as used in the previous study. In our study, the internal reliability was acceptable (α = 0.69).

#### 4.3.2 Experimental tasks

Participants in this study completed the same version of the *Social Inference task*, as described in Study 1 (see **Figure 1**). They also performed the *Classical conditioning task* (CC; 34, 35) as implemented in Study 1. Since the aim of this MEG study was to explore the electrophysiological counterpart of the therapeutic change induced by the CC intervention, only the positive and the neutral conditions were included.

### 4.4 Data acquisition and preprocessing

MEG data were recorded using a 306-channel (102 magnetometers and 204 planar gradiometers) system (Elekta^©^ VectorView; 1000 Hz sample rate, 0.01-330 Hz online filter) during the performance of the social inference task, both before and after the CC intervention. Electrooculogram and electrocardiogram channels were used to keep track of ocular and cardiac artefacts. The head shape was acquired using a three-dimensional Fastrak digitizer (Polhemus, Colchester, Vermont).

First, we used the spatiotemporal extension of the Signal Space Separation -tSSS- method (72) to remove those noises originated out of the head region. Afterwards, we used the package FieldTrip (73) for the automatic detection of ocular, cardiac, and muscular artefacts, and an independent component analysis based on SOBI (74) to eliminate the contribution of eye-and heart-related activity. Since the focus of this experiment was on participant’s reaction to the threatening faces (reaction stage of the social inference task), raw data was segmented into 1300ms epochs, spanning from 300 ms prior to the onset of the face and continuing for the 1000 ms that it remained on the screen. Epochs contaminated by artifacts were discarded for subsequent analyses. The 300 ms interval prior to the onset of the stimulus was used for baseline correction in each trial, and the resulting epochs were bandpass-filtered in the 1-30 Hz band. Further, the number of clean epochs included in the analysis was matched among conditions to prevent biases related to the amount of data. Because of the high redundancy in the MEG data after spatial filtering (97), only magnetometers’ data were used in the ERF analysis.

### 4.5 Data analysis

Based on the results obtained in the previous study (Study1) and given the exploratory and pilot nature of the study, the analysis was performed focusing on the Self condition.

Self-reported data: Firstly, the characteristics of the two classical conditioning groups were compared using Fisher’s exact test for categorical variables and Mann-Whitney’s U test for continuous variables. Secondly, as in Study 1, a measure of social inference was derived from the mean scores of the questions regarding evaluation and deservedness questions. Thirdly, to examine pre-post differences within groups, we used the Wilcoxon signed -rank test. Finally, to examine differences between groups, we used a Mann-Whitney U-test.

Neurophysiological data: The sets of clean epochs resulting from the preprocessing stage (see Data acquisition and preprocessing) were averaged to obtain an event-related field (ERF) for each participant and time (pre- and post-intervention), always using a minimum of 86 epochs. The focus of the study was on the two-way interaction between time and condition. To control for the familywise error rate in the context of multiple comparisons (multiple channels × time data points) we performed non-parametric, cluster-based, permutation statistics as implemented in Fieldtrip (73, 75). Since these cluster statistics are based on t-tests, we approached the analysis by calculating the individual pre/post-intervention change (subtracting the post-intervention from the pre-intervention neural responses) for each condition. This change was then compared between conditions using a two-sided independent samples comparison.

## 5 Results

### 5.1 Characteristics of the sample

There were no significant differences in the characteristics of the two groups at baseline measures (see **Table 5**) but the between-group age difference was notable (39 vs. 29 years) and approached significance.

**Table 5:** Demographic and clinical characteristics of group participants.

	Positive CC (*N* = 7)	Neutral CC (*N* = 5)	*U/F*	*p*
Demographic characteristics				
Age in years, mean (SD)	39.86 (7.29)	29.60 (9.12)	7.00	.09
Single status, n (%)	7 (100)	4 (80.0)	1.52	.21
Education, n (%)				
Primary School Secondary School College Education	4 (57.1) 0 (0) 3 (42.9)	2 (40.0) 2 (40.0) 1 (20.0)	2.92	.41
Employed, n (%)				
Unemployed Retirement	6 (85.7) 1 (14.3)	5 (100) 0 (0.0)	0.77	.37
Medication, n (%)				
Benzodiazepines Hypnotics (No benzo) Antipsychotics Anti-depressants Mood Stabilizers	2 (28.6) 2 (28.6) 7 (100) 5 (71.4) 2 (28.6)	0 (0.0) 0 (0.0) 5 (100) 1 (20.0) 0 (0.0)	1.71 1.71 __ 3.08 1.71	.19 .19 __ .08 .19
Clinical Characteristics				
Attachment (RQ)				
Anxious Attachment mean (SD) Avoidant Attachment mean (SD)	-0.71 (3.09) -1.00 (3.69)	0.00 (1.58) 1.20 (3.27)	15.0 11.50	.68 .32
Paranoia (PIQ), mean (SD)	18.42 (14.16)	16.0 (11.64)	15.5	.74
Symptomatology (HADS)				
Anxiety, mean (SD) Depression, mean (SD)	10.57 (2.50) 7.00 (4.35)	7.20 (3.83) 6.40 (2.07)	8.50 16.50	.14 .87
Self-esteem (RSES), mean (SD)	26.28 (6.10)	30.0 (2.54)	9.00	.16

CC, Classical Conditioning; HADS, Hospital Anxiety and Depression Scale; PIQ, Persecutory Ideation Questionnaire; RSES, Rosenberg self-esteem scale.

### 5.2 Pre-post changes in the social inference task after the CC intervention

Self-reported data: For the self-evaluation question, the within-group analysis revealed a marginally significant difference in the positive CC group with a large effect size (*r* = 0.71), indicating that the participants of the positive CC group had a more positive appraisal of themselves after the intervention. No significant differences were found between pre- and post-intervention in the neutral CC group, nor for the self-deservedness question in either the positive or the neutral CC groups. Between-group analysis revealed a possible marginal difference in the pre-self-evaluation question, but the effect size was small (PSest= 0.2). No significant differences between groups were found on the post-self-evaluation and self-deservedness questions (see **Table 6**).

**Table 6:** Means, standard deviations and non-parametric statistical tests for study differences in the social inference task.

A				
Social inference Task	Pre *M (SD)*	Post *M (SD)*	*Z*	*p*
Self-evaluation question				
Positive CC (*N*=7)	2.23 (0.20)	2.72 (0.68)	-1.89	.05*
Neutral CC (*N*=5)	2.53 (0.49)	2.54 (0.39)	-0.13	.89
Self- deservedness question				
Positive CC (*N*=7)	2.68 (0.61)	2.66 (0.72)	-0.16	.86
Neutral CC (*N*=5)	2.70 (0.35)	2.56 (0.48)	-0.94	.34
B				
Social inference Task	Positive CC (*N*=7) *M (SD)*	Neutral CC (*N*=5) *M (SD)*	*U*	*p*
Self-evaluation question				
Pre	2.23 (0.20)	2.53 (0.49)	7.00	.09
Post	2.72 (0.68)	2.54 (0.39)	16.50	.87
Self- deservedness question				
Pre	2.68 (0.61)	2.70 (0.35)	15.00	.68
Post	2.66 (0.72)	2.56 (0.48)	16.50	.87

A) Pre-post changes within groups in the social inference task; B) Differences between groups in the social inference task; CC, Classical Conditioning; Self-Evaluation question = *‘How do you think people have reacted to your entry?’*; Self-deservedness question = *‘Do you think you deserve this reaction?’*; M, Mean; SD, Standard Deviation.

**Figure 4:**
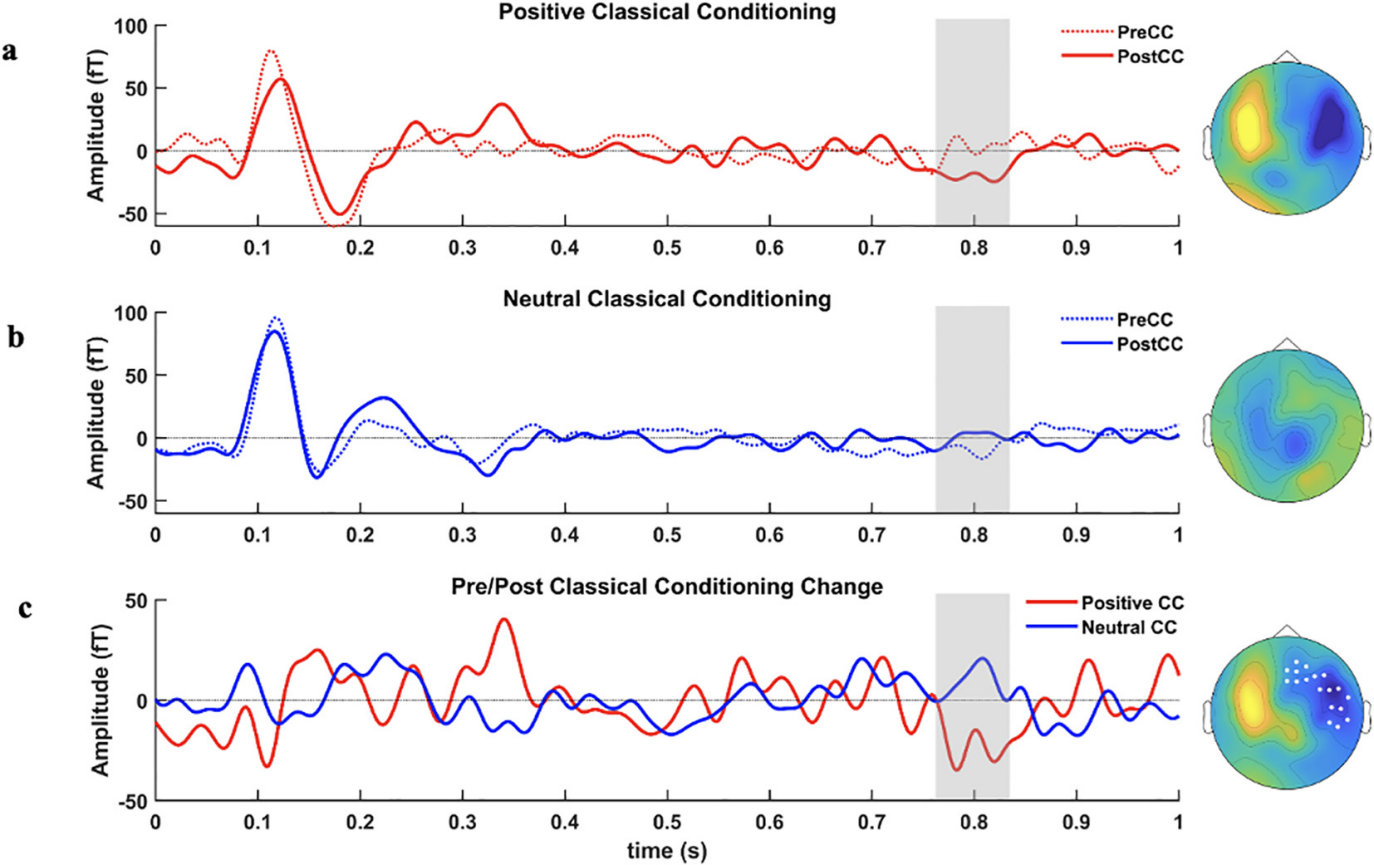
Sensor-space ERF waveforms. Grand average of ERF waveforms and their respective topographies for **(A)** pre-post differences in the positive CC group; **(B)** pre-post differences in the neutral CC group; and **(C)** pre-post change for positive and neutral CC groups. MEG sensors in the significant cluster (extending from 760 to 830 ms) are highlighted in white. For visualisation purposes, only sensors that contributed to the effect for at least 20 ms are depicted in the figure.

Neurophysiological data: Results from the non-parametric cluster-based comparisons of the pre-post changes between groups revealed a significant time × sensor cluster of differences, with a greater amplitude of change for the positive CC group (*p* < 0.01). This significant cluster encompassed 44 sensors with right topography and frontoparietal distribution, in a time window that emerged between 760 to 830 ms after the onset of the stimulus (see **Figure 4**). No significant differences were found between pre- and post-intervention neural responses, in either the positive or the neutral CC groups.

## 6 Discussion

The current study examined the effects of a novel brief evaluative conditioning intervention on social cognitive processes in nonclinical and clinical samples. To measure social cognitive processes, we also aimed to design a new ecologically valid task. Unlike traditional methods that rely on static images or written vignettes, our task presents scenarios from first- and third-person perspectives, which allow participants to engage in social judgements more naturally, resembling everyday social cognitive processes. Finally, we sought to determine the tolerability of the evaluative conditioning paradigm in patients with clinical paranoia and to provide a preliminary indication of whether the intervention affected patients’ social cognition at both the behavioural and neurophysiological levels.

Our main findings indicate that, after the evaluative conditioning intervention, participants made more positive evaluations of themselves on the social inference task. Interestingly, we found this effect in both our non-clinical and clinical samples, and the latter also showed associated neurophysiological changes during the social inference task. Our results also demonstrated relationships between baseline levels of paranoia, relevant psychological variables (i.e., anxious attachment and self-esteem) and evaluations of the self on the social inference task. Together, these preliminary results suggest the possibility that a brief intervention focusing on changing implicit self-esteem influences how individuals process social information that affects their self-concept.

### 6.1 The use of a new task to measure social cognition

Recent literature on paranoid beliefs has highlighted the need for more realistic tasks in which individuals make social judgements about themselves and others (8). Many studies that have used virtual reality have succeeded in measuring this effect. For example, Riches et al. (76) found that almost all participants reported social evaluative concerns and ideas of reference, including a perceived sense of being out of place, perceived antipathy of avatars, and the perception of being the object of discussion in scenes using virtual reality scenarios. However, a practical constraint is the availability of virtual reality technology. Therefore, we decided to design a new task that would allow us to measure social inference processes in an immersive way from a first- and third-person perspective without expensive technology. While we are aware of the existence of validated tests to measure social cognition (i.e., Hiting task, 77), and that these tests have been widely used in patients with paranoia (78), a practical limitation of these measures is that participants have to respond to static stimuli or interpret written social vignettes that are not representative of social cognitive processes in daily life (79). In this sense, some authors advocate the development of new validated tasks with psychometric and standardised data to measure social cognition (78). Although we recognise the importance of conducting these validation studies, our current study aimed to design a more ecological task to assess the effects of our brief evaluative conditioning intervention on social processes, and one that would also be feasible to use during MEG sessions with clinical patients.

Our results suggest that our task seems to be suitable for capturing paranoia’related phenomena. As in previous studies, we found that more levels of paranoia and anxious attachment were related to more negative evaluations of both targets (self and Gabriel). For example, Deng et al. (95) using a task to measure social interpretation bias and inflexibility, found that general population participants with higher levels of paranoia made more negative judgments of the characters involved in the scenarios and had more difficulty revising their initial interpretation, indicating a strong negativity bias in forming general impressions. Given that anxious attachment reflects an excessive need for approval, a negative self-image and a fear of rejection (80, 81), it is not surprising that our participants with higher levels of anxious attachment were more sensitive to social feedback and showed more negative interpretations of the scenarios. Previous studies have also shown that anxious attachment is related to a negative self-image (24, 82). Conversely, our results revealed that higher levels of self-esteem at baseline were associated with more positive evaluations of the self after the social feedback in the scenarios. These findings suggest that our task may be a valid and realistic method of measuring social cognition.

We failed to find any association between paranoia and paranoia-related variables (insecure attachment styles, anxiety, depression and self-esteem) and responses to the deservedness question. One possible explanation is that the non-clinical sample had low levels of paranoia, and we did not have a sufficient clinical sample or the measures to classify participants into ‘poor me’ and ‘bad me’ categories. Another possibility is that the question we employed failed to capture the complexity of deservedness estimations, which are highly unstable in paranoid patients (83).

### 6.2 Effects of the evaluative conditioning intervention

The main aim of this study was to explore the effects of an implicit evaluative conditioning paradigm on social cognitive processes. In addition, we also sought to determine whether this paradigm was tolerated by patients with clinical paranoia and feasible to use in clinical settings, providing a proof of concept indicating whether the intervention affects patients’ social cognition at the behavioural and neurophysiological levels. As we hypothesized, participants whose personal information was associated with positive stimuli in the evaluative conditioning intervention, subsequently made more positive self-evaluations on the social inference task. Encouragingly, we found this effect in both non-clinical and clinical participants. These results are in line with previous studies that have used the same evaluative conditioning parading and found changes in the implicit evaluations of the self and paranoid beliefs (34, 35). The fact that we only found changes in the self-evaluation question after the brief intervention and not in Gabriel’s evaluation question could be considered evidence that the implicit paradigm is aimed at modifying processes related to self-concept. Furthermore, in the non-clinical sample, our results revealed that after the positive intervention condition, participants showed reduced levels of anxious and avoidant attachment states, whereas paranoia increased after the negative CC condition. These results are further evidence of the relationship between implicit self-esteem or self-schemas and attachment styles and paranoia (12, 18). Our findings highlight the importance of considering these schemas as dynamic processes that can be modified as part of the therapeutic process (66, 84).

Despite the exploratory nature of this proof-of-concept study, at the neurophysiological level, as hypothesized, we found electrophysiological differences after the intervention with frontal topography generally associated with controlled processes. These electrophysiological differences were located in sensors covering the right frontoparietal cortex, in latencies (760-830ms) in which the Late Positive Potential (LPP) is commonly observed. This electrophysiological component is frequently related to emotional regulation strategies following pleasant and unpleasant images, facial expressions, or even threatening faces (85, 86), as well as in patients with schizophrenia in association with alterations in social cognitive processes (43). Interestingly, the LPP has also been commonly related to controlled, rather than automatic cognitive processes during emotion regulation (87), and the topography of the effect points to the engagement of medial and right sections of the prefrontal cortex, tightly associated with cognitive control and inhibitory processes (88, 89). Altogether, our results suggest that people with paranoia can reappraise social stimuli after a brief intervention. Our neurophysiological findings also align with those of Kumari et al. (48) and Mason et al. (90), which suggest greater integration between higher-order cognitive systems involved in information reappraisal within the social-affective task following a CBT intervention in paranoid patients. Our approach differed in the use of a brief evaluative classical conditioning intervention targeting implicit processes, rather than an explicit CBTp intervention.

The finding that we only detect a change in the self-evaluation question, and not in the self-deservedness question, could be attributed to several factors. First, as noted above, it is possible that our social inference task does not effectively capture this type of judgment, thus limiting our ability to discern alterations in it. Moreover, our evaluative conditioning intervention primarily targets implicit self-esteem closely associated with preverbal or implicit social attitudes, such as emotional and social perception, rather than more deliberative processes, such as attributional ones, in which individuals analyse the causes of social events (91).

### 6.3 Strengths and limitations

Despite the promising results of this proof-of-concept study, several limitations need to be acknowledged. Firstly, in the social inference task, we only used Caucasian male faces. This decision was made because the female stimuli in the database are often misperceived as male (i.e., they are bald and hairless faces) (27); hence, our results may not extend to female faces or faces from different ethnic backgrounds. This shortcoming has also been noted by other authors who have indicated that Western cultures have dominated social cognitive research, and few studies have made direct cross-cultural comparisons (79). In addition, the use of computer-generated faces as stimuli might have limited the ecological validity of our study. Additionally, we did not assess ethnicity, which could affect face perception, so our results may not extend to faces from different ethnic backgrounds. However, as a strength, we covaried by sex in Study 1 because the majority of the sample was female while the main character of the other scenes was Gabriel and all six faces shown were male. The low reliability of the insecure state attachment scales in our sample should also be considered when interpreting the results, and it may be due to our selection of only two items with the highest loading on each attachment factor. Regarding study 2, the results should be interpreted with caution, since the sample was small and included only men. Given that the cost of MEG prohibited comparing genders, we chose to include only men for two reasons. First, the highest prevalence of clinical diagnosis or severity of paranoia is found in men (92) and second, there might be behavioural and neurophysiological differences in emotional processing due to gender (93). Another limitation is the between-group age difference. While this difference did not reach statistical significance, with a larger sample size, it could have been controlled more effectively to reduce potential confounding effects. The small sample size also limits the generalisability of the behavioural and MEG results. Additionally, one limitation affecting the comparability between both studies is that we used different measures of attachment and paranoia in Study 1 and Study 2. Although it would have been preferable to use the same measures across both studies, in the clinical sample, we prioritised shorter questionnaires with good validity to ensure participants, who had active symptomatology and were undergoing a long session, could complete the evaluation.

Nonetheless, our results suggest that even a brief evaluative conditioning intervention can have measurable effects in both nonclinical and clinical populations, opening the door for further research with more intensive interventions.

### 6.4 Clinical implications and conclusions

The studies reported here represent the very earliest stages in the development of an approach to treating paranoid symptoms which is novel and, to our knowledge, completely unlike any other psychological intervention for psychosis. Whereas conventional psychotherapeutic techniques target, by necessity, explicit reasoning processes, we chose to try and manipulate implicit cognition. The results are encouraging, suggesting that implicit cognition is manipulable, that doing so affects underlying neurophysiological mechanisms, and that there may be an impact on paranoid symptoms. However, much more work is required to discover whether this approach can be adapted to produce meaningful clinical change, and can be delivered in routine clinical settings. In addition, future studies could also explore the effects of this intervention on other relevant psychological constructs, such as explicit self-esteem, which were not addressed in the current study but may provide valuable insight into the comprehensive treatment of paranoia. Further research must also establish the magnitude of the effects and their durability following different doses of the procedure, exploring the longer-term impact of the intervention to determine whether repeated sessions could lead to trait-level changes in paranoia and attachment schemas similar to approaches like Attachment-focused iMAgery therapy. Additionally, any complex psychological phenomenon such as paranoia will likely involve elements of both implicit and explicit cognition. Hence, even if clinical effectiveness can be demonstrated, our approach may be most useful when combined with more traditional CBTp approaches rather than when used as an alternative to them.

## Data Availability

The original contributions presented in the study are included in the article/supplementary material, further inquiries can be directed to the corresponding author/s.
